# Supplementary material for: Control theory illustrates the energy efficiency in the dynamic reconfiguration of functional connectivity
Source: Commun Biol. 2022 Apr 1;5:295. doi: 10.1038/s42003-022-03196-0 (PMC8975837; doi:10.1038/s42003-022-03196-0)
Supplement: Supplementary file 2 — Supplementary Information [file 42003_2022_3196_MOESM2_ESM.pdf]

Supplementary Information for

Control Theory Illustrates the Energy Efficiency in the Dynamic Reconfiguration of Functional Connectivity

Shikuang Deng, Jingwei Li, B.T. Thomas Yeo, and Shi Gu*

Shi Gu*
Email: gus@uestc.edu.cn

Supplementary Note 1

S1. Regional Spatial Distribution of Functional Controllability. Here we illustrate the regional distribution of the average and modal controllability map (Fig. S1). The average functional controllability values are calculated across all subjects. The regional distribution results of dFC-Autoregression are similar to the results of dFC-Slidingwindow, since they both reflect the controllability of dynamic functional connectivity. Then we give the student's t-test results (Table. S1 and S2) of the system-wise controllability measurements (Fig. 2 a and b in the main text) for the three cases.

Fig. S1 Regional distribution of the average and modal controllability maps.

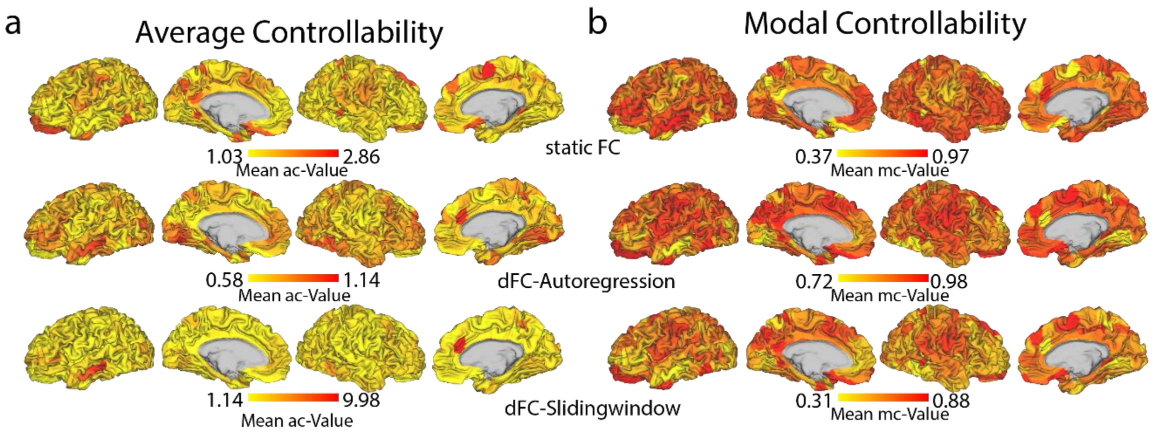

We illustrate the regional distribution of the average (**a**) and modal controllability (**b**) map. The controllability values are averaged across all subjects.

	Static FC VS. dFC-Autoregression		Static FC VS. dFC-Slidingwindow		dFC-Autoregression VS. dFC-Slidingwindow	
	T value	p-value	T value	p-value	T value	p-value
Visual	34.66	5.85e-230	-38.90	2.64e-287	-75.53	1.56e-377
Somatomotor	53.37	3.41e-326	-13.02	4.93e-62	-83.00	1.64e-402
Dors_Attn	59.38	3.27e-411	-45.68	4.01e-340	-97.43	2.74e-474
Salience	105.97	5.95e-573	-8.52	3.75e-23	-96.10	4.11e-478
Limbic	94.90	9.42e-517	-5.34	5.36e-10	-82.89	3.54e-423
Fronto_Pariental	44.42	1.46e-300	-106.34	6.25e-615	-143.28	7.96e-633
Default	72.24	1.22e-496	-64.34	2.17e-429	-115.15	3.86e-537
Sub_cor	96.47	2.24e-504	48.02	2.85e-411	-43.43	1.86e-217

Table. S1. *Student's t-test results (N = 865) of the system-wise average controllability for three cases (w.r.t Fig 2. a).*

	Static FC VS. dFC-Autoregression		Static FC VS. dFC-Slidingwindow		dFC-Autoregression VS. dFC-Slidingwindow	
	T value	p-value	T value	p-value	T value	p-value
Visual	-30.79	2.42e-225	32.54	4.26e-294	195.78	8.61e-763
Somatomotor	-61.66	7.92e-438	3.83	2.58e-7	194.60	3.92e-760
Dors_Attn	-59.62	4.54e-492	38.27	1.47e-353	292.99	2.29e-1051
Salience	-121.96	4.04e-744	-10.69	2.13e-41	296.54	1.17e-1138
Limbic	-115.88	5.26e-728	-17.33	5.73e-97	232.40	2.38e-1064
Fronto_Pariental	-32.88	2.37e-232	99.56	1.12e-864	310.78	2.10e-1301
Default	-66.21	1.83e-565	57.94	1.36e-560	326.58	2.08e-1208
Sub_cor	-142.06	5.69e-704	-69.78	4.80e-578	145.23	1.05e-641

Table. S2. *Student's t-test results (N = 865) of the system-wise modal controllability from three cases (w.r.t. Fig 2. b).*

S2. Measurement robustness. We select those subjects whose four sessions' resting fMRI data and cognitive data are valid and calculate their graph and control theoretical measurements on each session. For each subject, we obtain $\binom{4}{2} = 6$ correlations between the results from any two different sessions for each type of measurement. Then we compute the correlation's mean and standard deviation across all individuals. In Fig. S2, the upper triangular elements record the results for sFC-Correlation, and the lower triangular elements are the results for dFC-Autoregression. These results verified the repeatability of the adopted graph and control theoretical measurements across different sessions. One observation here is that the repeatability of the control measurement is higher than the graph measurement, especially for the dFC-Autoregression.

Fig. S2. The control and graph measurements similarity across four sessions.

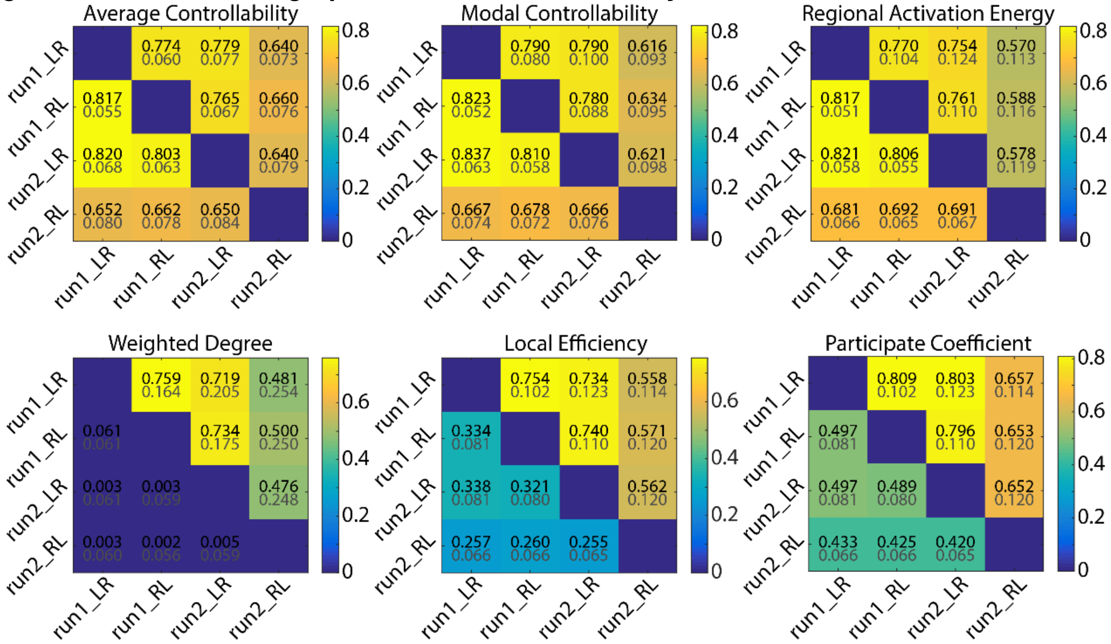

For any subject, we calculate the control measurement and graph measurement under 4 sessions and calculate the similarity of the 4 results. Then we give the mean value and standard deviation of the correlation of all subjects. We use a matrix to display the results, the upper triangle is the result from the static FC, and the lower triangle results from the dFC-Autoregression.

S3. Prediction Results for All Cognitive Scores. Here we provide the prediction results of all 58 cognitive scores from session run1_LR (Fig. S3). The Kernel Ridge model with cosine kernel is used as in the main context. Then we set four column vectors composed of 58 prediction results from four sessions and compute their pairwise correlation (Fig. S4). The stability of prediction results from the control theoretical measurement (0.771±0.076) is higher than that from the graph theoretical measurement (0.732±0.051).

Fig. S3. Prediction of 58 cognitive scores.

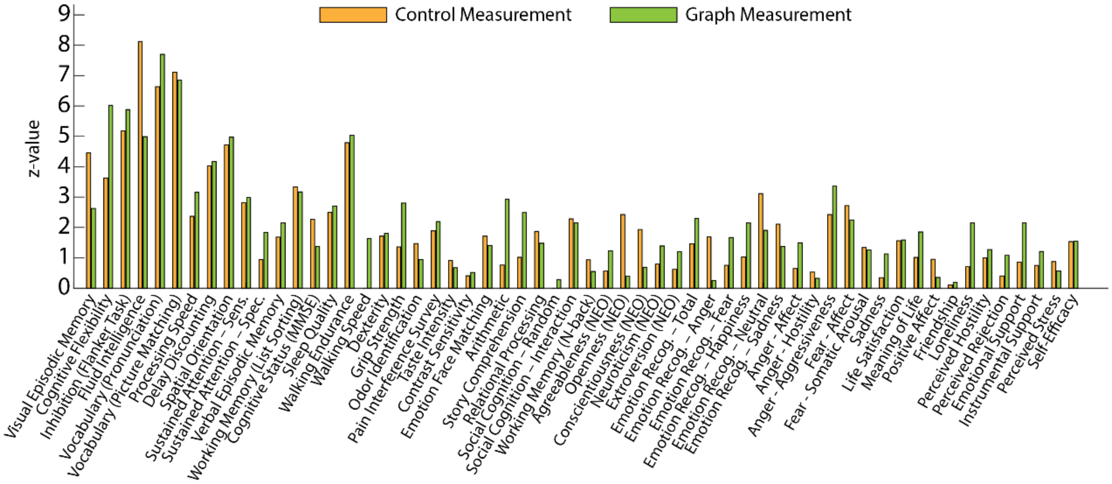

This figure shows all the results using control measurement and graph measurement to predict 58 cognitive scores in the session run1_LR. We use the same Kernel Ridge model with cosine kernel same as the main text. The prediction results of control measurement and graph measurement are different in most of the scores.

Fig. S4. The prediction similarity of four sessions with cosine kernel.

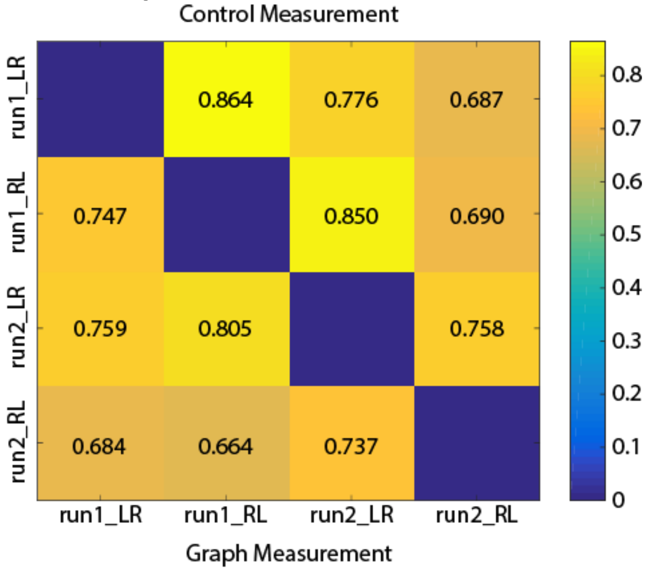

Here we show the similarity of the four sessions' prediction results. The upper and lower triangles of the matrix indicate the similarity of the 58 cognitive scores' prediction results by control measurement and graph measurement, respectively. This figure also shows that compared to graph measurement (0.732±0.051), control measurement (0.771±0.076) is more stable in four sessions.

S4. Prediction Results using Linear Kernel. We replace the cosine kernel in the prediction model by the linear kernel and examine the dependence of results on the kernel choice (Fig. S5). Most of the cognitive scores that can be predicted with the cosine kernel remain predictable with the linear kernel. However, the repeatability of the correlations between the observed and predicted scores calculated through linear kernel has a decrease compared to that through the cosine kernel, especially for the graph measurement (Fig. S6).

Fig. S5. Prediction of 58 cognitive scores with linear kernel.

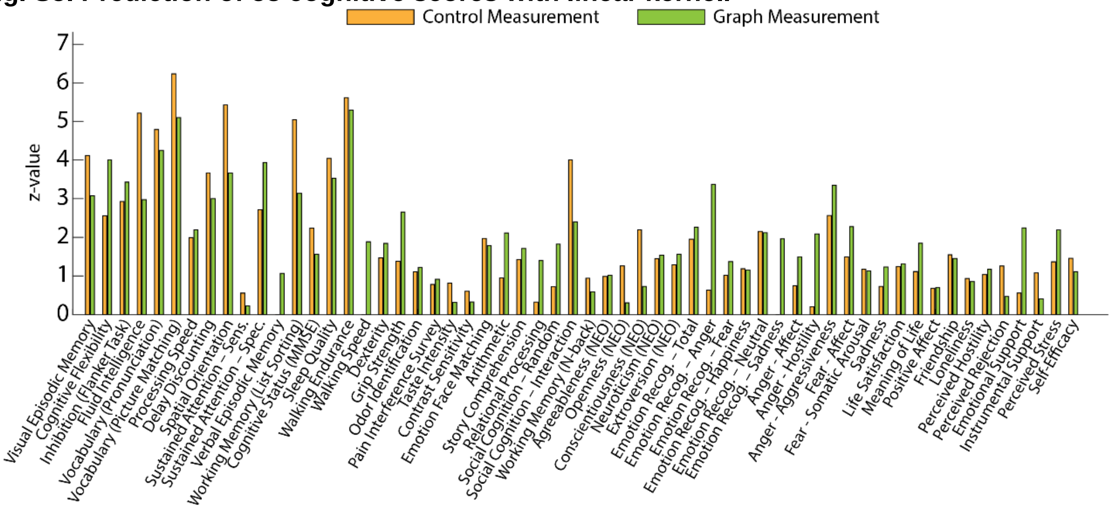

We change the model kernel to a linear kernel and predict the 58 cognitive scores. The result is almost the same as the result of the situation using the cosine kernel. The dominance recognition scores of the control measurement and graph measurement have not changed much, but the degree of superiority has changed.

Fig. S6. The prediction similarity of four sessions with linear kernel.

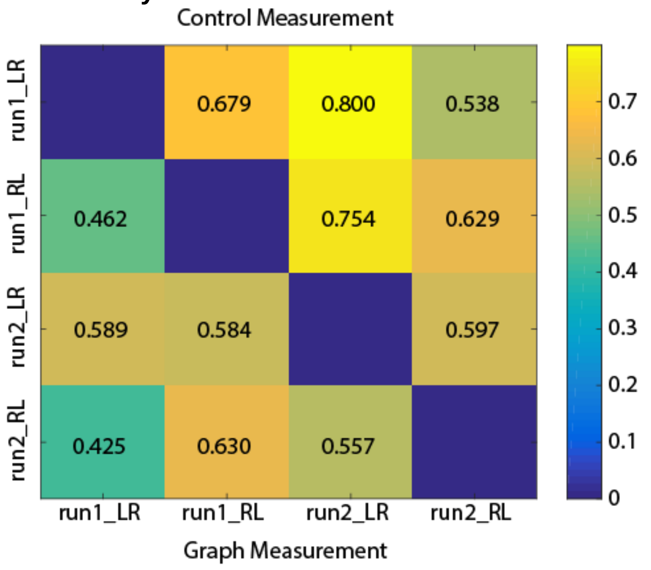

We also give a matrix to show the result of the prediction results similarity of four sessions. Regardless of the control measurement (upper triangle 0.666±0.098) and graph measurement (lower triangle 0.541±0.080), all matrix positions have decreased, indicating that the repeatability of the linear kernel will reduce.

S6. Energy Efficiency Explains the Dynamic Reconfiguration of Functional Connectivity.
In Fig. 3 b,c,d, we plot the variance range to demonstrate the phenomenon. Here we further provide with the one-tailed t-test result for Fig.3 b (t=83.78, p=8.65e-1288, $N = 10380$), c (t=207.36, p=7.55e-4491, $N = 10380$), and d (t=165.34, p=3.49e-3462, $N = 10380$) to verify our statement that the dynamic control paradigm is more efficient than that of static control. Next, we change the initial and final state from the mean value of the center range of the initial and final snapshots to the BOLD value of the initial and final moments (Fig. S7). Compared with the results in Fig. 3 of the main text, the conclusion is not changed. And the one-tailed t-test result also demonstrates that dynamic control energy consummation is less than the result of static control under the case of the driven nodes of DM (*t=22.27, p=3.32e-87,* $N = 865$), DM+DA+SA+FP (*t=89.37, p=8.34e-439,* $N = 865$), and the whole brain (*t=148.75, p=6.73e-618,* $N = 865$).

Fig. S7. Energy Efficiency Explains the Dynamic Reconfiguration of Functional Connectivity.

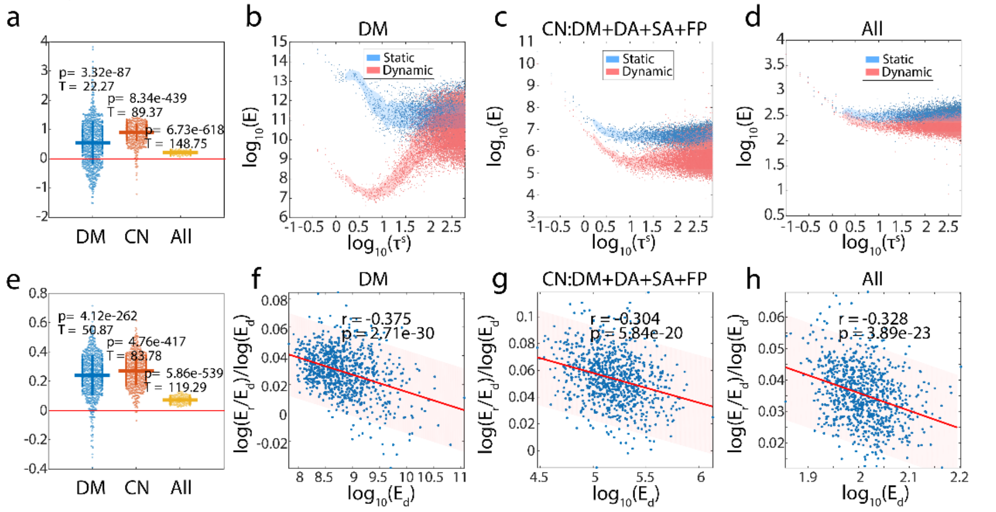

DM: Default Mode, DA: Dorsal Attention, SA: Salience, FP: Fronto-parietal, CN: Combined Network, All: Whole Network

As an alternative to the results shown in Fig. 3, we change the initial state to the first time point BOLD vector and the final state to the last time point BOLD vector of the stime series. (a-d) Dynamic control paradigm is energetically more efficient than that of static control no matter we set the control set as Default Mode Network (DM), the combination of Default Mode, Dorsal Attention (DA), Salience (SA), and Fronto-parietal networks (FP), or the whole network (All). (e) The driving energy cost will increase when the order of snapshots is disrupted. (f-h) The relative increase of log-energy after shuffling the snapshot order is negatively related to the log-energy of dynamic control consummation.

	$\tau^S < 10$		$10 \leq \tau^S < 100$		$\tau^S \geq 100$		$\tau^S > 0$	
	T value	p-value	T value	p-value	T value	p-value	T value	p-value
DM	97.2	2.14e-188	66.66	1.90e-492	89.5	6.06e-979	99.53	7.61e-1422
DM,DA,SA,FP	54.44	3.02e-36	121.01	2.57e-827	260.24	2.29e-4195	289.85	1.48e-4964
ALL	29.02	3.70e-08	147.41	1.80e-638	346.58	2.05e-3513	369.92	2.91e-3837

Table. S3. *Student's t-test results of the energy efficiency comparison within different intervals of control time (Fig. 3 b-d).*
