# Supplementary material for: Control theory illustrates the energy efficiency in the dynamic reconfiguration of functional connectivity
Source: Commun Biol. 2022 Apr 1;5:295. doi: 10.1038/s42003-022-03196-0 (PMC8975837; doi:10.1038/s42003-022-03196-0)
Supplement: Supplementary file 3 — Description of Additional Supplementary Files [file 42003_2022_3196_MOESM3_ESM.pdf]

Description of Additional Supplementary Files

File name: Supplementary Data 1
Description: It provides the average controllability (sheet 1 corresponds to Fig. 2a) and modal controllability (sheet 2 corresponds to Fig. 2a) of regions that belong to each system.

File name: Supplementary Data 2
Description: Sheet 1 gives the nodes value of log10 (E_static/E_dynamic) (Fig. 3 a) and log10(E_shuffled-order/E_dynamic) (Fig. 3 e). Sheet 2 provides logarithmic energy values in Fig. 3 b-d. Sheet 3 provides the values for each point in the scatterplot (Fig. 3 f-h).

File name: Supplementary Data 3
Description: Sheet 1 gives the best prediction z-values for the control and graph. Sheet 2 gives the z-value decrement after remove a region or a system for drawing the Fig. 4.

File name: Supplementary Data 4
Description: It corresponds to Fig. 5 and provides the best prediction z-value from control measurements, graph measurements and combine measurements.
