# Supplementary material for: Control theory illustrates the energy efficiency in the dynamic reconfiguration of functional connectivity
Source: Commun Biol. 2022 Apr 1;5:295. doi: 10.1038/s42003-022-03196-0 (PMC8975837; doi:10.1038/s42003-022-03196-0)
Supplement: Supplementary file 8 — Reporting Summary [file 42003_2022_3196_MOESM8_ESM.pdf]

nature research

Corresponding author(s): Shi Gu

Last updated by author(s): Nov 28, 2021

Reporting Summary

Nature Research wishes to improve the reproducibility of the work that we publish. This form provides structure for consistency and transparency in reporting. For further information on Nature Research policies, see our Editorial Policies and the Editorial Policy Checklist.

Statistics

For all statistical analyses, confirm that the following items are present in the figure legend, table legend, main text, or Methods section.

n/a	Confirmed	
☐	☒	The exact sample size (n) for each experimental group/condition, given as a discrete number and unit of measurement
☐	☒	A statement on whether measurements were taken from distinct samples or whether the same sample was measured repeatedly
☐	☒	The statistical test(s) used AND whether they are one- or two-sided<br>*Only common tests should be described solely by name; describe more complex techniques in the Methods section.*
☐	☒	A description of all covariates tested
☐	☒	A description of any assumptions or corrections, such as tests of normality and adjustment for multiple comparisons
☐	☒	A full description of the statistical parameters including central tendency (e.g. means) or other basic estimates (e.g. regression coefficient) AND variation (e.g. standard deviation) or associated estimates of uncertainty (e.g. confidence intervals)
☐	☒	For null hypothesis testing, the test statistic (e.g. F, t, r) with confidence intervals, effect sizes, degrees of freedom and P value noted<br>*Give P values as exact values whenever suitable.*
☒	☐	For Bayesian analysis, information on the choice of priors and Markov chain Monte Carlo settings
☒	☐	For hierarchical and complex designs, identification of the appropriate level for tests and full reporting of outcomes
☒	☐	Estimates of effect sizes (e.g. Cohen's d, Pearson's r), indicating how they were calculated

Our web collection on statistics for biologists contains articles on many of the points above.

Software and code

Policy information about availability of computer code

Data collection	We used the Human Connectome Project 1200S release, which is a public dataset. http://www.humanconnectomeproject.org/
Data analysis	The analyses were performed with Matlab and Python.

For manuscripts utilizing custom algorithms or software that are central to the research but not yet described in published literature, software must be made available to editors and reviewers. We strongly encourage code deposition in a community repository (e.g. GitHub). See the Nature Research guidelines for submitting code & software for further information.

Data

Policy information about availability of data

All manuscripts must include a data availability statement. This statement should provide the following information, where applicable:

- Accession codes, unique identifiers, or web links for publicly available datasets
- A list of figures that have associated raw data
- A description of any restrictions on data availability

The used data were public online.

Field-specific reporting

Please select the one below that is the best fit for your research. If you are not sure, read the appropriate sections before making your selection.

☒ Life sciences ☐ Behavioural & social sciences ☐ Ecological, evolutionary & environmental sciences

For a reference copy of the document with all sections, see nature.com/documents/nr-reporting-summary-flat.pdf

Life sciences study design

All studies must disclose on these points even when the disclosure is negative.

Sample size	N = 865.
Data exclusions	One frame before and two frames after them are also removed. Discard the remaining segments containing less than five frames as well as runs with > 50% of censored frames.
Replication	We replicate the main findings based on different runs of scans and report them in the supplement.
Randomization	n/a
Blinding	n/a

Reporting for specific materials, systems and methods

We require information from authors about some types of materials, experimental systems and methods used in many studies. Here, indicate whether each material, system or method listed is relevant to your study. If you are not sure if a list item applies to your research, read the appropriate section before selecting a response.

Materials & experimental systems

n/a	Involved in the study	
☒	☐	Antibodies
☒	☐	Eukaryotic cell lines
☒	☐	Palaeontology and archaeology
☒	☐	Animals and other organisms
☐	☒	Human research participants
☒	☐	Clinical data
☒	☐	Dual use research of concern

Methods

n/a	Involved in the study	
☒	☐	ChIP-seq
☒	☐	Flow cytometry
☐	☒	MRI-based neuroimaging

Human research participants

Policy information about studies involving human research participants

Population characteristics	Please refer to HCP's website.<br>https://www.humanconnectome.org/study/hcp-young-adult/document/1200-subjects-data-release
Recruitment	Please refer to HCP's website.<br>https://www.humanconnectome.org/study/hcp-young-adult/document/1200-subjects-data-release
Ethics oversight	Please refer to HCP's website.<br>https://www.humanconnectome.org/study/hcp-young-adult/document/1200-subjects-data-release

Note that full information on the approval of the study protocol must also be provided in the manuscript.

Magnetic resonance imaging

Experimental design

Design type	resting state
Design specifications	n/a
Behavioral performance measures	The behavioral scores provided by the HCP release. Some are based on the executive performance. Some are self-

Behavioral performance measures | reported scores.

Acquisition

Imaging type(s): functional

Field strength: 3T

Sequence & imaging parameters: Every subject has four 14.4 min runs (1200 frames) of resting-state functional series, which have a temporal resolution of 0.72s. TE = 33.1ms. flip angle = 52 deg. FOV: 208x180mm, Slice Thickness: 2.0mm; 72 slices; 2.0mm isotropic voxels. Echo spacing: 0.58ms, BW: 2290Hz/Px. Check https://www.humanconnectome.org/storage/app/media/documentation/s1200/HCP_S1200_Release_Reference_Manual.pdf for the detail.

Area of acquisition: whole brain

Diffusion MRI ☐ Used ☒ Not used

Preprocessing

Preprocessing software: The fmriPrep was used.

Normalization: Resting-state fMRI data is projected to the fs_LR surface space by the multimodal surface matching method (MSM 525 ALL).

Normalization template: MNI152

Noise and artifact removal: Then ICA-FIX method is used to clean the imaging data, including the regression of 24 motion-related parameters (6 classical motion parameters, their derivatives, and the squares of these 12 parameters).

Volume censoring: Frames with FD> 0.2 mm or DVARS > 75 are removed by motion censoring. One frame before and two frames after them are also removed. Discard the remaining segments containing less than five frames as well as runs with > 50% of censored frames.

Statistical modeling & inference

Model type and settings: Pearson's correlation.

Effect(s) tested: The correlation between the variables of interest was used to determine whether the predicted effect was significantly correlated with the observed effect.

Specify type of analysis: ☒ Whole brain ☐ ROI-based ☐ Both

Statistic type for inference (See Eklund et al. 2016): n/a

Correction: n/a

Models & analysis

n/a	Involved in the study
☐	☒ Functional and/or effective connectivity
☐	☒ Graph analysis
☐	☒ Multivariate modeling or predictive analysis

Functional and/or effective connectivity: We used the Pearson's correlation for functional connectivity and auto-regression coefficients for the effective connectivity.

Graph analysis: The weighted graph is used.

Multivariate modeling and predictive analysis: The kernel ridge regression method was used for the prediction model and the performance was estimated through 10-fold cross-validation.
